# 

**Published:** 1875-04

**Authors:** 


### Contents of April Number.

### ORIGINAL DEPARTMENT.

ART. I.—Clinical Remarks upon Surgical Cases. By Prof. J. F. Miner, M. D. Reported by W. W. Miner, M. D. .......................... 321
ART. II.—Abstract of the Proceedings of the Buffalo Medical Association, March 2, 1875. Reported by E. N. Brush, M. D., Secretary pro tem... 327
ART. III.—Camp Diarrhœa and Dysentery. By T. M. Johnson, M. D.... 330

### MISCELLANEOUS.

Note on Salicylic Acid. By E. R. Squibb, M. D............................ 337
Cure of Femoral Aneurism, treated first by Compression, and subsequently by Ligation of the External Iliac. By C. C. F. Gay, M. D........... 344
Report of a Case of Inversio-Uteri, of two years standing. Reduced by Taxis. By B. F. Lawson, M. D......................................... 349
Resection of a portion of the Continuity of the Ulna and Radius for ununited Colles Fracture.............................................. 353

### EDITORIAL DEPARTMENT.

American Medical Association .......................................... 354
Medical Literature,.................................................... 355
Obituary. Rev. Asher Wright,............................................ 356
Items,................................................................. 356

BOOKS REVIEWED:

Compendium of Children's Diseases. By Dr. Johann Steiner,... 358
Cyclopædia of Practice of Medicine,.............................. 358
Diseases of the Stomach. By Wilson Fox,.......................... 359
Dental Pathology and Surgery. By S. James A. Salter,......... 360

Books and Pamphlets Received,............................................ 360

THE BUFFALO

